# Correction: Todorov et al. Electromagnetic Sensing Techniques for Monitoring Atopic Dermatitis—Current Practices and Possible Advancements: A Review. *Sensors* 2023, *23*, 3935

**DOI:** 10.3390/s23249888

**Published:** 2023-12-18

**Authors:** Alexandar Todorov, Russel Torah, Mahmoud Wagih, Michael R. Ardern-Jones, Steve P. Beeby

**Affiliations:** 1 Centre of Flexible Electronics and E-Textiles, School of Electronics and Computer Science, University of Southampton, Southampton SO17 1BJ, UK; 2 James Watt School of Engineering, University of Glasgow, Glasgow G12 8QQ, UK; 3 Clinical Experimental Sciences, Faculty of Medicine, University of Southampton, Southampton SO16 1DU, UK

## Addition of an Author

**Mahmoud Wagih** was not included as an author in the original publication [1].

## Additional Affiliation

2. James Watt School of Engineering, University of Glasgow, Glasgow G12 8QQ, UK

## Author Contributions Correction

The corrected Author Contributions statement is given below. 

**Writing—original draft preparation, A.T.; writing—review and editing, M.R.A.-J., R.T., M.W. and S.P.B.; supervision, M.R.A.-J., R.T., M.W. and S.P.B. All authors have read and agreed to the published version of the manuscript.**

The authors state that the scientific conclusions are unaffected. This correction was approved by the Academic Editor. The original publication has also been updated.
